# Strengthening Africa's response to Mpox (monkeypox): insights from historical outbreaks and the present global spread

**DOI:** 10.1016/j.soh.2024.100085

**Published:** 2024-10-28

**Authors:** David B. Olawade, Ojima Z. Wada, Sandra Chinaza Fidelis, Oluwafemi S. Oluwole, Chibuike S. Alisi, Nifemi F. Orimabuyaku, Aanuoluwapo Clement David-Olawade

**Affiliations:** a Department of Allied and Public Health, School of Health, Sport and Bioscience, University of East London, London, United Kingdom; b Department of Research and Innovation, Medway NHS Foundation Trust, Gillingham ME75NY, United Kingdom; c Department of Public Health, York St John University, London, United Kingdom; d Division of Sustainable Development, College of Science and Engineering, Hamad Bin Khalifa University, Qatar Foundation, Doha, Qatar; e School of Nursing and Midwifery, University of Central Lancashire, Preston Campus, United Kingdom; f Department of Public Health, School of Health and Life Science, Teesside University, Middlesbrough, United Kingdom; g English Programme, Bowen University, Iwo, Osun State, Nigeria; h Endoscopy Unit, Glenfield Hospital, University Hospitals of Leicester, NHS Trust, Leicester, United Kingdom

**Keywords:** Mpox, Zoonotic diseases, Outbreak management, Emerging infectious diseases, Surveillance systems

## Abstract

Mpox, formerly known as Monkeypox, is a viral zoonotic disease endemic to Central and West Africa that has posed significant public health challenges since its identification in 1970. Despite decades of experience in managing outbreaks, the 2022–2024 Mpox outbreaks exposed substantial gaps in global preparedness and response, leading the World Health Organization (WHO) to declare a Public Health Emergency of International Concern (PHEIC) in 2022. The resurgence of cases in Europe in 2022 and the more recent emergence of the virulent clade Ⅰb in the Democratic Republic of the Congo (DRC) in 2024 have highlighted a critical need for improved proactive and response strategies to curb the epidemic. This narrative review examines the historical and recent epidemiology of Mpox in Africa and explores the factors that have limited effective management. These include objective influences such as viral mutations, zoonotic transmission patterns, and environmental changes like deforestation, as well as subjective factors, including delayed responses, limited vaccine availability, cessation of smallpox vaccinations, and inequitable access to healthcare. In particular, the review emphasizes the ongoing disparities in global health equity, as wealthier nations have been able to secure vaccines and therapeutics quickly, while endemic regions in Africa continue to struggle with limited resources. The review also discusses how socio-economic and cultural factors, combined with weak public health infrastructure and inadequate surveillance systems, perpetuate cycles of outbreak in vulnerable populations. Furthermore, the emergence of clade Ⅰb in 2024, with its higher virulence and mortality rates among children, particularly in rural areas, underscores the urgency of addressing the evolving epidemiological landscape of Mpox. In response to these challenges, this review recommends strengthening healthcare infrastructure, enhancing surveillance systems, ensuring equitable access to vaccines and treatments, and integrating environmental management into public health strategies. Global collaboration remains essential to provide African countries with the resources and support needed to manage and prevent future outbreaks effectively. Without these measures, the world risks a prolonged public health crisis with far-reaching consequences for both Africa and the global community.

## 1 Introduction

Mpox is a viral zoonotic disease that has garnered increasing attention due to its rising incidence and public health implications, particularly within the tropical rainforest regions of Central and West Africa [1,2]. The Mpox virus (MPXV) causes the disease, a member of the genus *Orthopoxvirus*, which is also responsible for causing smallpox (variola virus) [[3], [4], [5]]. According to the World Health Organization (WHO) [6], Mpox has two main clades: clade Ⅰ and clade Ⅱ. Clade Ⅰ has subclades Ⅰa and Ⅰb and clade Ⅱ has subclades Ⅱa and Ⅱb, where subclade Ⅱb is known to have caused a global outbreak in 2022–2023, while subclades Ⅰa and Ⅰb still pose health risks in 2024 as common symptoms include skin rashes, mucosal lesions, fever, headaches, and muscle aches [7]. Table 1 highlights some key differences between both clades. Vaccination, alongside other public health measures, is advised. Although Mpox is generally considered less severe than smallpox [8,9], it remains a significant concern in public health, especially in regions where it is endemic [4,10]. The disease manifests with symptoms like smallpox, including fever, rash, and lymphadenopathy, but with generally lower mortality rates [3]. However, the morbidity associated with Mpox, coupled with its potential for human-to-human transmission, underscores its importance as a persistent and emerging infectious disease threat [11,12].

**Table 1:** Differences between Mpox clades.

	Mpox characteristics				References
Clades	Ⅰ		Ⅱ		[13]
Subclades	Ⅰa	Ⅰb	Ⅱa	Ⅱb	[13]
Regions	Central and East Africa	Central and East Africa	West Africa	Global outbreak	[13]
First reported case	1970 (the Democratic Republic of the Congo)	2023 (Kamituga, South Kivu, the Democratic Republic of the Congo)	1971 (Nigeria)	2017 (Nigeria)	[13,14]
Mortality	High (up to 10 %)	5%–10 %	Low (<1 %)	0.2%–3.6 %	[[15], [16], [17]]
Transmission	Mostly via zoonoses (rodents)	Human transmission	Mostly via zoonoses	Human transmission	[15,17]
Symptoms	Mimics smallpox presentation; symptoms include fever, headache, and malaise followed by 2–3 weeks centrifugal rash and synchronous lesion	Pus-filled blisters, muscle and back aches, headache, swollen lymph nodes	Significant prodromal phase with fever, vesicular lesions and generalized rash development	No prodomomes; fever, localized rash with lesions, frequent inflammation with oral presentation with or without pharyngitis	[17]
Susceptibility	Children under 10 years old	Sexually active adults (20–40 years)	Young men (20–40 years)	Men infected with HIV and men who have sex with men	[15,17]
Virulence	Highest after Ⅰb	Highest	Lower than Ⅰb	Lowest	[15,16]
Route of infection	Mostly household transmission	Physical contact, sexual contact, aerosol vehicle	Predominantly via close sexual contact	Sexual contact	[16,17]

The first documented case of human Mpox was reported in 1970 in the Democratic Republic of the Congo (DRC), shortly after the global eradication of smallpox was declared [12]. This initial case marked the beginning of what would become a recurrent public health challenge in Africa. Over the decades, Mpox has caused numerous outbreaks, predominantly in countries such as the DRC, Nigeria, Cameroon, and the Central African Republic [[18], [19], [20]]. These outbreaks varied in size and impact, but they collectively highlight the ongoing struggle to control and manage the disease in regions where it is endemic. The cessation of the smallpox vaccination program in 1980, following the successful eradication of the disease, inadvertently created a population that is increasingly susceptible to other orthopoxviruses, including Mpox [12,21]. The smallpox vaccine, which provided cross-protection against Mpox, was no longer administered, leading to a gradual decline in population immunity [22]. This has likely contributed to the observed increase in Mpox cases over the past few decades [22]. Additionally, the lack of routine vaccination, combined with other socio-economic and environmental factors, has facilitated the resurgence of Mpox, raising concerns about its potential to cause larger and more widespread epidemics [4,11,23,24].

Recent years have seen a marked increase in Mpox outbreaks, with significant events occurring in Nigeria in 2017 and 2019 [21], as well as the ongoing endemic situation in the DRC [25,26]. These outbreaks have been characterized by higher transmission rates, more severe clinical presentations, and an increasing number of cases involving human-to-human transmission [11,12,[27], [28], [29]]. The 2022 global outbreak, which saw the virus spread to non-endemic regions such as Europe and North America, further underscored the growing threat of Mpox as a global public health issue [30,31]. This situation has led to heightened awareness and concern among public health officials, researchers, and policymakers worldwide [[32], [33], [34]]. Despite the growing recognition of Mpox as a significant public health challenge, many African countries continue to face difficulties in effectively managing and controlling outbreaks such as the situation in the DRC [35]. These challenges are multifaceted, involving weak public health infrastructure, inadequate surveillance systems, limited access to vaccines and therapeutics, and significant socio-economic and cultural barriers [18,19,36]. Additionally, environmental and ecological changes, such as deforestation and increased human–animal interactions, have further complicated efforts to control the disease [23,37].

In 2024, a significant outbreak of Mpox emerged, particularly affecting the DRC and several other African countries [26]. The outbreak, driven primarily by the clade Ⅰ strain of the virus, has been declared a Public Health Emergency of International Concern (PHEIC) by the WHO due to its rapid spread and high fatality rate, which ranges between 3% and 4% [38]. The global response to this Mpox outbreak involved coordinated efforts from various international health organizations, including the WHO and the Africa Centres for Disease Control and Prevention (Africa CDC). This resurgence of Mpox highlights the ongoing public health challenges in managing infectious diseases, particularly in regions with limited healthcare infrastructure [39]. The ongoing struggle to manage and control Mpox outbreaks in African countries presents a significant public health challenge that has been exacerbated by rising incidence rates, inadequate healthcare infrastructure, and limited access to critical resources such as vaccines and antiviral treatments [40,41]. The cessation of smallpox vaccination programs and the resulting decline in population immunity [3,42], coupled with socio-economic and environmental factors, have contributed to the resurgence of Mpox in regions where it remains endemic [21,43]. The problem is further complicated by the spread of the virus to non-endemic regions, as seen in the 2022 global outbreak [2] and the recent 2024 outbreaks in sub-Saharan Africa [44], underscoring the need for a comprehensive understanding of the factors driving these outbreaks. The rationale for this narrative review is to address the gaps in knowledge regarding the multifaceted challenges that hinder the effective management of Mpox in Africa. By analyzing historical and current outbreaks, the review seeks to identify the underlying issues that contribute to the persistence of the disease. The primary objective of this review is to provide a detailed examination of the reasons behind Africa's ongoing struggles with Mpox, to highlight the lessons that need to be learned, and to propose strategies for improving public health responses to prevent future outbreaks from escalating into larger epidemics.

## 2 Methods

### 2.1 Literature search and selection

This narrative review was conducted through a comprehensive literature search to gather relevant information on the challenges faced by African countries in managing Mpox outbreaks. The primary sources of data included peer-reviewed journal articles, reports from public health organizations such as the WHO and the Africa CDC, as well as relevant books, conference papers, and governmental publications. The literature search was performed using multiple electronic databases, including PubMed, Scopus, Google Scholar, and Web of Science, to ensure a wide range of sources were considered. Key search terms used in the literature search included “Mpox”, “outbreak management”, “public health infrastructure”, “Africa”, “zoonotic diseases”, “vaccination”, “surveillance”, “epidemiology”, “Nigeria”, “Democratic Republic of Congo”, “global health”, and “disease control”. Boolean operators (AND, OR) were applied to combine these terms and refine the search results.

### 2.2 Inclusion and exclusion criteria

The selection criteria for literature in this narrative review emphasized relevance and quality. A total of 112 references were selected for inclusion, spanning the period from 1970 to 2024, focusing on historical and contemporary developments of Mpox, particularly in Central and West Africa, where the disease is endemic. The review prioritized studies on Mpox outbreaks, public health responses, disease management challenges, and factors affecting transmission and control. Excluded were studies unrelated to Mpox or those focusing on non-African regions unless they offered relevant comparative insights. Non-English articles were excluded unless translated versions were available, and opinion-based publications lacking empirical data or rigorous analysis were also omitted to uphold scholarly integrity. This process ensured that only high-quality sources, reflective of the changing epidemiological landscape and public health interventions, were included in the review, allowing for a comprehensive and reliable evaluation of Mpox outbreaks.

### 2.3 Data extraction and synthesis

Data extraction aimed at identifying key themes related to the challenges of Mpox outbreaks in Africa. Systematic collection of information focused on areas such as public health infrastructure, surveillance systems, vaccine and treatment access, socio-economic and cultural factors, and environmental influences. The gathered data were organized thematically to help synthesize information and identify existing patterns and gaps in the literature. The synthesis involved summarizing findings from various studies and reports, comparing different viewpoints, and integrating them into a cohesive narrative. The goal was to provide a comprehensive overview of Mpox management in Africa, highlight challenges and contributing factors, and suggest recommendations for improving outbreak control and prevention.

## 3 Historical background and epidemiology in Africa

**Fig. 1:**
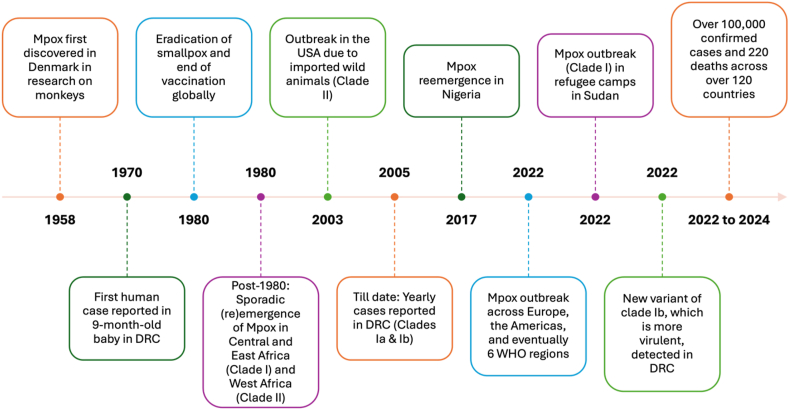
Timeline of Mpox from discovery till recent outbreaks. Abbreviation: DRC: the Democratic Republic of the Congo; WHO, World Health Organization.

The historical background and epidemiology of Mpox reveal the disease's evolving nature and management challenges. Early outbreaks were sporadic and limited to rural areas with poor healthcare, but recent cases have increased in frequency, severity, and urban spread. The 2022 and 2024 global outbreaks emphasized the need for better surveillance, healthcare infrastructure, and international cooperation to prevent larger epidemics [[45], [46], [47]]. Understanding Mpox's history and trends is essential for addressing current management challenges and potential global health risks. Fig. 1 provides a comprehensive timeline of Mpox incidences from its discovery to the present, illustrating the major outbreaks as reported by WHO. The first human case of Mpox was identified in 1970 in the DRC, formerly known as Zaire [14,48,49]. This initial case emerged shortly after the successful eradication of smallpox, a closely related orthopoxvirus [50]. Concerns were raised due to the clinical similarities between Mpox and smallpox, including fever, rash, and lymphadenopathy [4,51]. However, unlike smallpox, Mpox was primarily zoonotic, with most early cases linked to direct contact with infected animals, particularly rodents and primates [23]. Throughout the 1970s and 1980s, Mpox outbreaks were sporadic and largely confined to remote, rural areas in Central and West Africa [18,52]. Countries such as the DRC, Nigeria, Cameroon, Central African Republic, Liberia, and Sierra Leone reported cases, although the true extent of the disease was likely underrecognized due to weak healthcare infrastructure and inadequate surveillance systems [53]. These early outbreaks were characterized by low human-to-human transmission, with most cases resulting from zoonotic exposure [54]. The limited spread within human populations was partly due to the relative isolation of affected communities and the lack of efficient transmission pathways.

In recent years, Mpox has reemerged as a significant public health threat, with outbreaks becoming more frequent, widespread, and severe, especially in the Central and West African regions [55,56]. The resurgence of Mpox has been particularly notable since the early 2000s, with a marked increase in both reported cases and the geographic range of outbreaks [31,57]. This trend has raised concerns about the potential for Mpox to cause larger and more sustained epidemics [58]. One of the most significant outbreaks in recent years occurred in Nigeria in 2017, marking the country's first Mpox cases in nearly four decades [21]. This outbreak is an introduction of the clade Ⅱb variant, which saw over 300 suspected cases across multiple states, was notable for its high rate of human-to-human transmission [59] and the involvement of urban populations, a departure from previous outbreaks that had primarily affected rural areas [27]. The 2017 Nigerian outbreak also exposed limitations in the country's public health infrastructure, including challenges with case detection, insufficient testing capacities, and delays in contact tracing and isolation [60,61].

**Fig. 2:**
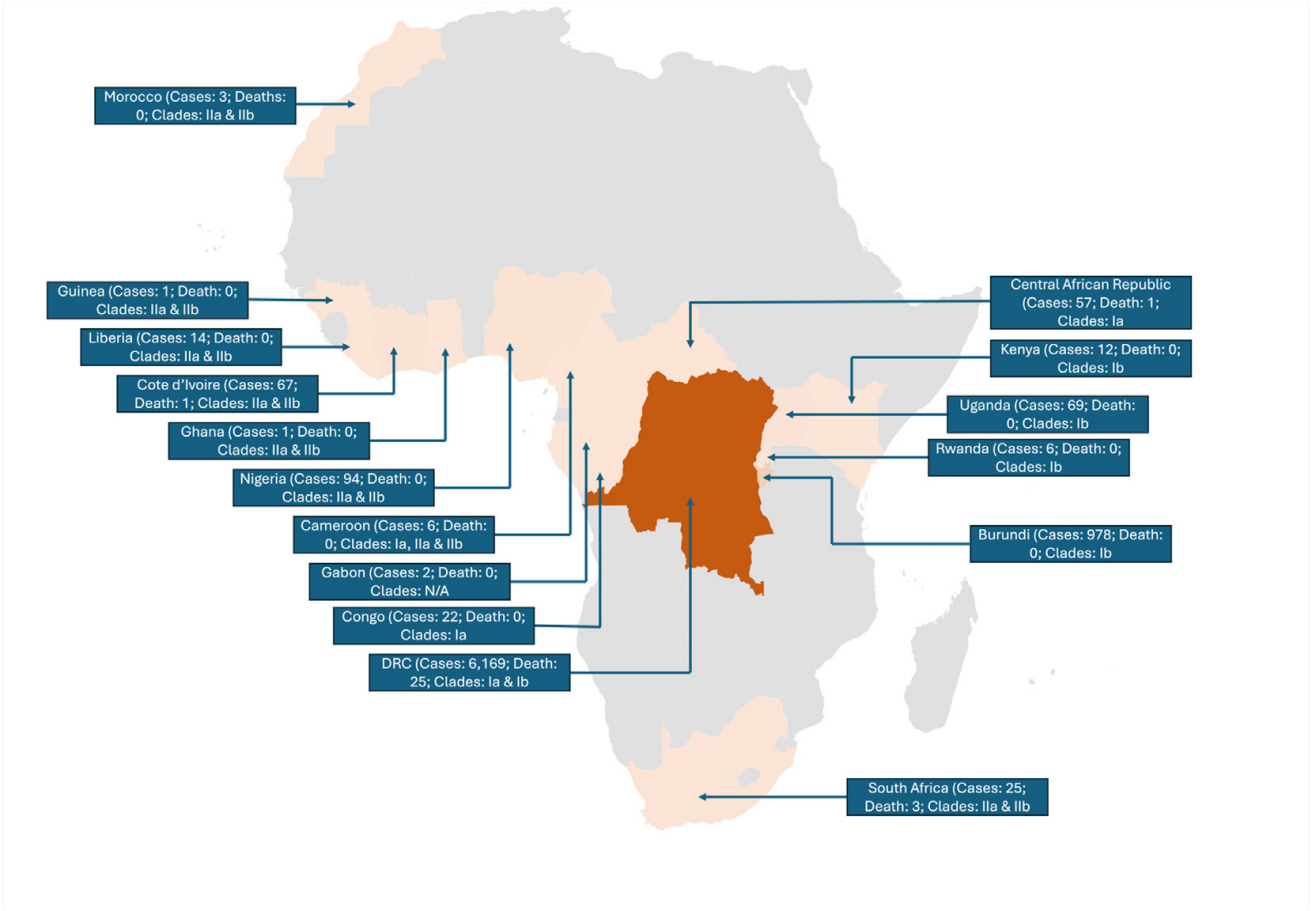
Map of Africa highlighting the cases, mortality, and clades associated with the 2024 Mpox outbreak from January 1st to October 6th based on WHO data [6]. Abbreviation: DRC: the Democratic Republic of the Congo; WHO, World Health Organization. Note: the map was designed by the authors and data captured in the map is adapted from the cited source.

The DRC remains a significant hotspot for Mpox outbreaks, with the country accounting for a substantial proportion of reported cases in Africa [12,62]. Ongoing conflict and political instability in the DRC have further complicated public health efforts, contributing to the persistence and spread of the virus [63]. Additionally, the DRC's dense forests continue to serve as a reservoir for the virus within wildlife populations [64]. The 2022–2024 global Mpox outbreaks marked a turning point in the virus's epidemiology, as cases spread beyond endemic regions to non-endemic countries in Europe, North America, and Asia [57,65]. However, the cases reported in the African continent are low compared to others. This is a suggestion of the limited testing capacity of African countries even in Mpox hotspots [66]. These outbreaks were characterized by high transmission rates and severe clinical presentations, particularly in populations with no prior exposure to the virus [28,67]. Vulnerable populations, especially children under five, experienced a disproportionate number of cases and fatalities during the 2024 outbreak [68], further highlighting the global implications of Mpox [69]. Table 2 highlights historical Mpox data across Africa, while Fig. 2 shows the number of Mpox cases, mortality, and associated clades in 2024 across Africa.

**Table 2:** Historical timeline of significant Mpox outbreaks in Africa.

Country	Year	Confirmed cases	Deaths	Response	Challenge
Burundi	2024	8	0	Rapid diagnostic testing, contact tracing and treatment, isolation [70]	Logistics, poor education, and rural location of cases
Cameroon	1979	1	0	Case isolation and contact tracing	Limited awareness, the remote area has poor access to healthcare and lack of infrastructure for sample testing, regional conflict, and loss of social order [71,72]
	1989	1	0		
	2018–2021	9	0		
	2022–2024	35	5		
Central African Republic	1984	6	0	Emergency alert of central healthcare bodies, contact tracing and laboratory testing [73]	Poor awareness about Mpox, rural areas have limited infrastructure for specimen collection and sampling [72]
	2001	3	2		
	2010	1	0		
	2012	2	0		
	2015–2021	93	10		
	2022–2024	58	1		
Congo	2003	11	1	Collaboration with international partners, isolation, contact tracing and surveillance	Conflict-affected areas, geographical isolation, and lack of public health capacity
	2009	2	0		
	2017	7	6		
	2019	2	0		
	2022–2024	24	1		
Cote d’Ivoire	1971	1	0	Contact tracing and quarantine of confirmed cases	Limited infrastructure for surveillance and management [72]
	1981	1	–		
Democratic Republic of the Congo	1970–1980	48	–	Laboratory diagnostic, improved regional capacity in managing outbreaks, surveillance and contact tracing [74]	Lack of access to PCR instruments for diagnosis, use of viable but unreliable alternatives for case confirmation [66,72,74]
	1981–1986	338	33		
	1987–1995	–	–		
	1996–2004	>200 per year	–		
	2005–2015	>1000 per year	–		
	2016–2021	Unspecified	483		
	2022–2024	>3500	453		
Gabon	1987	5	2	Immediate contact tracing, contact isolation	Coordination challenges and limited infrastructure [72]
Ghana	2022–2024	131	4	Contact tracing, isolation and surveillance	Limited infrastructure for testing, treatment and management of cases [72]
Liberia	1970	4	0	Contact tracing, isolation and rapid testing	Limited health infrastructure, conflict and war [75]
	2017	2	0		
	2022–2024	18	0		
Nigeria	1971	2	0	Organisation of response team, contact isolation, quarantine arrangements [76]	Poor infrastructure for laboratory testing for Mpox confirmation [72,76]
	2017–2021	226	9		
	2022–2024	867	9		
Sierra Leone	1970	1	1	Contact tracing, isolation and testing through PCR instruments [77]	Early detection and diagnosis due to mimicry of other diseases [77]
	2014	1	0		
	2017	1	0		
	2019	1	0		
	2021	1	0		
South Africa	2022–2024	27	3	Contact tracing, surveillance and isolation	Poor awareness of Mpox, logistics in management of handling cases [72]
South Sudan	2005	10	0	Contact tracing and isolation	Conflict and internal displacement in affected areas, limited healthcare infrastructure [72]

Abbreviation: CDC, Centres for Disease Control and Prevention.

**Fig. 3:**
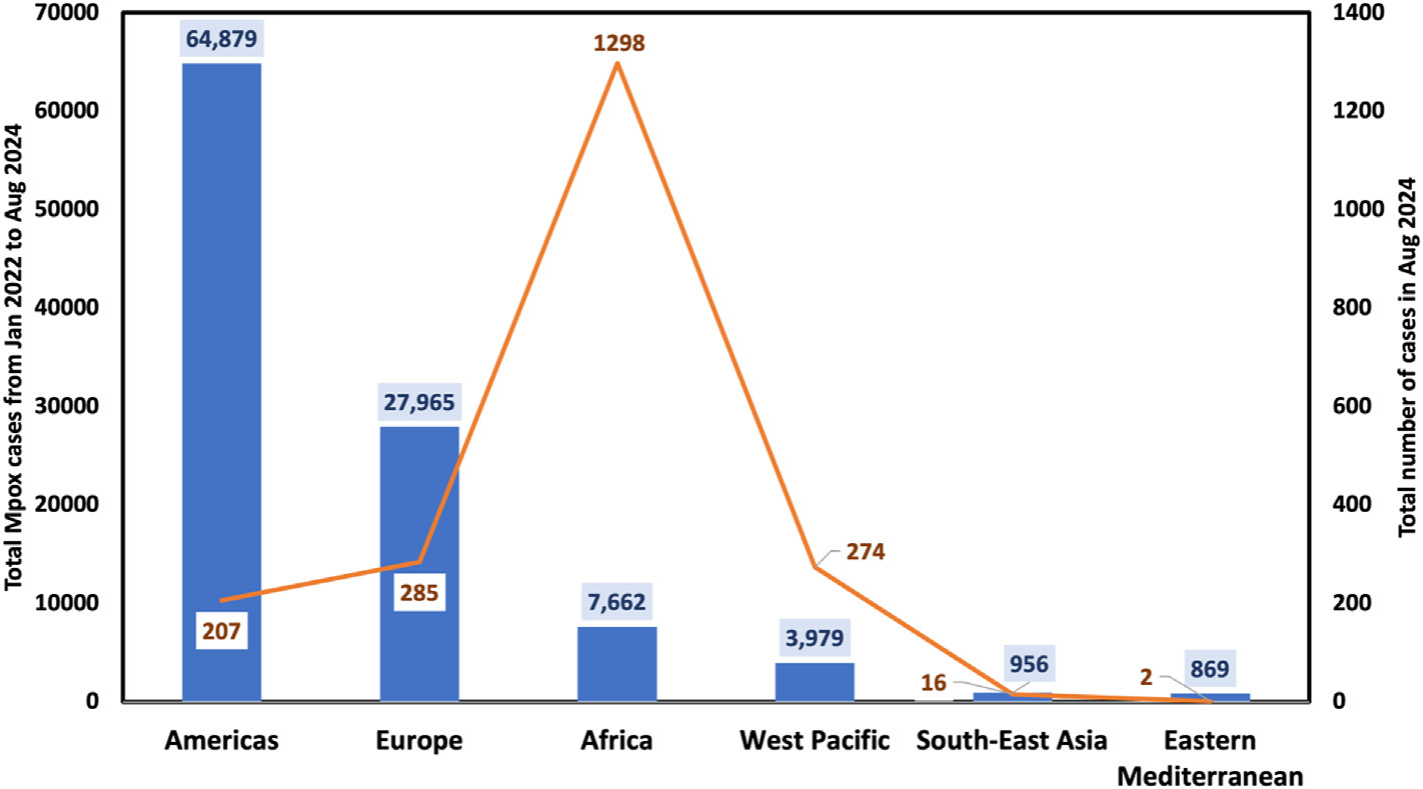
Global Mpox cases among WHO regions from January 2022 to August 2024 [6,7]. Abbreviation: WHO, World Health Organization. Blue bars represent the total Mpox cases from Jan 2022 to Aug 2024; orange line graph represent the total number of cases in Aug 2024 in different WHO regions.

On a global scale, there are disparities in Mpox case numbers between Africa and other WHO regions. According to data from the WHO [6,7], a substantial shift in the geographic distribution of Mpox cases occurred over time. By August 2024, a rise in new cases was observed, with Africa accounting for 62.3 % of cases reported in the past month, signalling a growing outbreak in the region. Conversely, during the earlier peak of the global outbreak in 2022, Mpox cases were heavily concentrated in countries outside Africa, particularly in the United States (33,812 cases), Brazil (12,206 cases), and several European nations like Spain, France, and the United Kingdom. The shift in case distribution highlights the divergent trajectories of the Mpox epidemic. While the initial global surge in 2022 affected the United States and European regions most heavily, African countries, particularly the DRC, are now experiencing a heightened burden. In the past year, the African region reported the highest number of cases (5688) and deaths (34) compared to other WHO regions, with the Americas (4729 and 9, respectively) and the Western Pacific Region (2483 and 10, respectively) following closely. Fig. 3 shows the total cases among WHO regions between January 2022 and August 2024 and the cases for August 2024, indicating a shift in global trends. This evolving landscape reinforces the need for global cooperation in Mpox response efforts, particularly as Africa continues to experience a growing share of the global burden.

### 3.1 Genetic typing and mutation sites in MPXV

The MPXV exhibits significant genomic variation, particularly with its recent outbreaks, highlighting the need to understand its evolutionary trajectory. Genetic typing of the MPXV has revealed the presence of distinct lineages that have evolved over time. These include the previously dominant West African clade and the Central African clade, with the emergence of new lineages such as C.1.1 and B.1, which have been pivotal in recent outbreaks [78]. The C.1.1 lineage, diverging from the C.1 lineage, marks a significant evolutionary event, characterized by numerous missense mutations and increased apolipoprotein B mRNA-editing catalytic polypeptide-like 3 (APOBEC3)-related mutations. These mutations, particularly the lineage-defining APOBEC3-related mutation that disrupts the *N2L* gene, a viral innate immune modulator, are thought to drive the virus's adaptability and transmissibility, significantly impacting its pathogenic evolution [78].

Further, the B.1 lineage of the MPXV, which has been responsible for global outbreaks since 2022, contains key non-synonymous mutations that have altered the virus's transmissibility and pathogenicity. Phylogenetic analyses have identified 49 substitutions, with 23 of these classified as non-synonymous mutations in the viral genome, some of which have been attributed to the APOBEC3 mutational pattern. Class Ⅰ variants, specifically, have been linked to changes in viral protein conformation, affecting the virus's characteristics and its interactions with host cells [79]. Roychoudury et al. [80] also affirmed that the B.1 lineage was primarily attributed to the 2022 outbreak in Washington, USA. This study analyzed 109 viral genomes from clinical specimens collected between July and August 2022, revealing low genetic diversity within the B.1 lineage, with sublineages such as B.1.1, B.1.2, B.1.3, B.1.4, and B.1.8 indicating multiple independent introductions into the region. Genomic analysis identified 138 unique single nucleotide polymorphisms (SNPs) across the viral genome, resulting in 66 mutations, including amino acid substitutions and deletions in 51 genes. Notably, five unique amino acid substitutions—S553N, A1232V, D1546N, D1604N, and S1633L—were found in the surface glycoprotein OPG210, while three mutations—E306K, D441Y, and E553K—were located in OPG189, a gene encoding an ankyrin-repeat protein involved in viral–host interactions [80]. These mutations suggest potential changes in viral fitness and immune evasion capabilities.

**Fig. 4:**
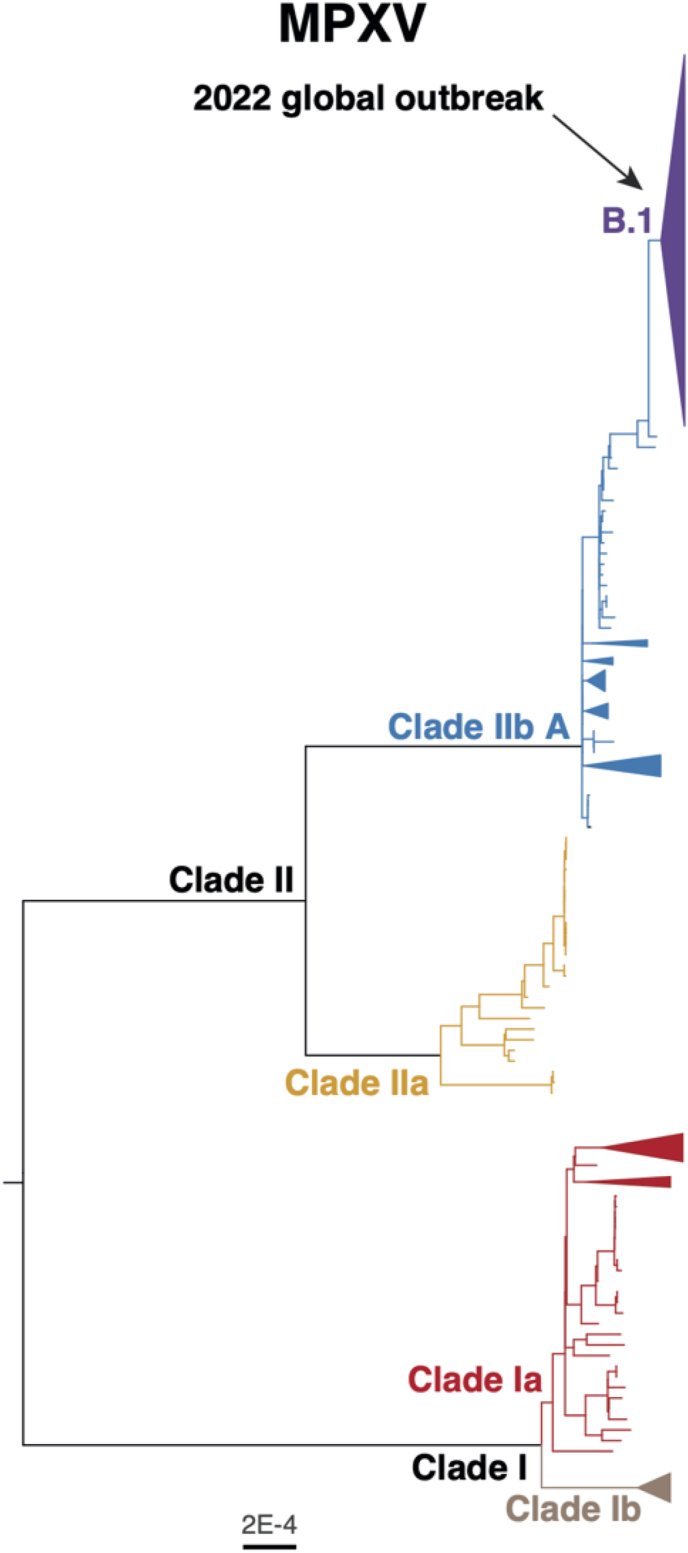
Phylogeny of all Mpox virus (MPXV) clades. (adapted from WHO, 2024; Available at https://worldhealthorg.shinyapps.io/mpx_global/).

This suggests that the MPXV, much like other poxviruses, has evolved mechanisms to evade host immune responses while maintaining viral fitness. Interestingly, mixed viral populations within patients, as seen with single nucleotide variants (SNVs), further highlight the virus's intra-host genetic diversity. This diversity raises the possibility of co-infection with slightly divergent strains, which could complicate disease management and treatment strategies [81]. The accumulation of mutations across the MPXV's evolutionary history is not just a reflection of genetic drift but may also suggest viral adaptation to different environments and hosts. Studies comparing the recent MPXV sequences with other orthopoxviruses, such as cowpox and variola (smallpox), reveal a high degree of sequence conservation. However, recent mutations affecting key viral proteins involved in immune evasion suggest that the MPXV is continually evolving, driven by selective pressures within hosts [82]. By integrating these findings, we can underscore the necessity of continuous genomic surveillance to track the evolutionary trajectories of MPXV strains, identify emerging mutations, and better inform public health strategies. This, in turn, reinforces the critical role of genetic typing and mutational analysis in understanding the spread and potential pathogenicity of Mpox. Fig. 4 provides the phylogeny of the MPXV as provided by WHO (WHO, 2024).

### 3.2 Influencing factors in Mpox outbreaks in Africa

#### 3.2.1 Objective factors

Before 2022, Mpox outbreaks were predominantly restricted to Central and West African nations, including the DRC, Nigeria, and Cameroon [14,19]. These outbreaks were largely zoonotic in nature, with humans contracting the virus through direct contact with infected animals such as rodents and primates. This is a critical environmental factor that influenced the epidemic's progression, as rural populations in these regions engaged in activities like hunting, handling, or consuming bushmeat, which increased their risk of exposure [83]. Human-to-human transmission was limited and occurred primarily in household settings.

Another significant objective factor is the global interconnectedness that facilitated the spread of the virus outside endemic areas. In 2003, an outbreak in the United States occurred following the importation of infected exotic animals from Ghana [84,85]. This event highlighted how international trade and travel could serve as pathways for the virus to reach non-endemic regions, though these cases were rare before 2022. By 2022, the virus had spread significantly in non-endemic regions, particularly through human-to-human transmission, primarily in men who have sex with men [59,86]. The outbreak in these populations marked a significant shift in transmission dynamics, driven by close skin-to-skin contact, particularly during sexual activity. Such changes in transmission patterns reflect the adaptive capacity of the virus, which, when coupled with mutations, demonstrates its potential to spread through various social and environmental contexts.

Human activities such as deforestation, agricultural expansion, and urbanization have led to greater human encroachment into wildlife habitats, increasing the risk of spillover events in Mpox with zoonotic transmission. As people come into closer contact with wildlife reservoirs of the virus, particularly rodents and primates, the likelihood of transmission from animals to humans has grown. This dynamic is particularly evident in regions like Central Africa, where the destruction of natural habitats has brought humans into more frequent contact with animals that carry the virus [28,64]. Additionally, climate change has contributed to the alteration of wildlife distributions, further complicating the dynamics of Mpox transmission. Changes in temperature, rainfall patterns, and ecological conditions may influence the spread of both animal reservoirs and the virus, increasing the likelihood of future outbreaks [23].

Moreover, viral mutations, such as the emergence of clade Ⅰb in 2024, further complicated the epidemic. Clade Ⅰb was first detected in the DRC in June 2022, which has since been associated with a surge in cases in 2024 [6]. Compared to other Mpox subtypes, this clade has been linked to a higher mortality rate, especially among children, particularly affecting children under 15 years old, who account for 68 % of reported cases and 85 % of deaths [70]. Clade Ⅰb has been associated with a mortality rate as high as 10 %, significantly higher than the 3 % mortality rate typically observed with other clade I strains [87]. Moreover, the presentation of the disease in clade Ib cases is distinct, with rashes covering the entire body, in contrast to other strains where lesions are often confined to the mouth, face, and genitals [87]. As of mid-August 2024, the DRC had reported over 16,800 cases, with the vast majority of global Mpox cases and deaths occurring in this country. The outbreak had expanded across 23 of the DRC's 26 provinces, with children under five years old being particularly vulnerable, making up a significant proportion of the cases and fatalities [7]. Since Clade Ib was first identified in DRC in June 2022, it has been associated with recurring outbreaks in the DRC, with case numbers remaining under 100 until April 2024, when a significant increase was observed. By September 2024, cases surged to a peak of 300, marking a critical turning point in the outbreak dynamics of clade Ib [6]. Unlike the 2022–2024 outbreak, which primarily impacted men who have sex with men (MSM) populations in urban settings, the Ib outbreak has displayed different epidemiological characteristics [88]. WHO reports indicate that the Ib variant is largely zoonotic, with transmission patterns similar to those seen in earlier African outbreaks [13]. However, it has also shown some human-to-human transmission, particularly within household settings. One key difference noted in WHO's epidemiological bulletins is that the Ⅰb variant has disproportionately affected children and rural populations, with fewer cases reported among MSM [13]. This suggests that the Ⅰb variant may be less transmissible through sexual networks compared to the Ⅱb variant that drove the 2022–2024 outbreak. The higher prevalence among children may be due to increased exposure to infected animals in rural areas or the fact that children are less likely to have been vaccinated for smallpox, which provides cross-protection against Mpox.

These findings underscore the need for heightened surveillance and response strategies, especially in regions where clade Ⅰb is spreading. The rapid increase in cases and their detection in countries outside of Africa raise concerns about the potential for wider transmission, highlighting the critical importance of understanding the transmission dynamics and clinical manifestations of this subtype. The Ⅰb outbreak in Africa has highlighted the ongoing challenges faced by countries in the region in managing zoonotic diseases [88].

#### 3.2.2 Subjective factors

Subjective factors, particularly related to public health response and preparedness, also played a significant role in the epidemic's spread. One of the most significant influencing factors has been the cessation of smallpox vaccination programs after the largescale eradication of smallpox even in the African continent [89,90]. The smallpox vaccine provided cross-protection against other orthopoxviruses, including MPXV. When vaccination programs were discontinued, the population's immunity to orthopoxviruses gradually declined. Younger populations, especially those born after 1980, now lack this cross-immunity, making them more susceptible to MPXV infection. This has created a larger pool of vulnerable individuals, which may have contributed to the resurgence of Mpox cases in recent decades. The increased susceptibility to Mpox has made outbreaks more difficult to contain, as the virus can spread more easily among unvaccinated populations [4,42,91]. A shortage of vaccines and treatments in endemic areas like the DRC further exacerbated the situation, leading to higher mortality rates and greater difficulties in controlling the outbreak.

Another key subjective factor was the delayed global response to the outbreak. Early outbreaks in Africa received limited international attention, and resources for outbreak management in endemic countries were often inadequate. Despite WHO efforts to improve local surveillance and manage zoonotic transmission in resource-limited settings, such as rural Africa, these outbreaks were often sporadic and localized. The lack of coordinated global action prior to 2022 allowed Mpox to remain a regional issue, with little investment in long-term preventive strategies, such as vaccination campaigns. For example, the virus spread outside endemic regions in 2022, and the international response was reactive rather than proactive. WHO declared Mpox a PHEIC in July 2022, but by then, the virus had already spread rapidly across Europe, North America, and other regions. The rapid dissemination of Mpox vaccines highlighted existing disparities in healthcare systems globally. Wealthy countries were able to quickly secure vaccine supplies, leaving many African nations struggling to manage their local outbreaks. The cessation of routine smallpox vaccinations globally after the disease was declared eradicated in 1980 also left a large portion of the global population susceptible to Mpox, particularly in Africa, where resources for vaccination campaigns were scarce [89]. Additionally, the emergence of clade Ⅰb in 2024, a more virulent strain associated with higher mortality rates, particularly among children, exposed gaps in global preparedness and coordination. WHO reports indicate that the Ⅰb variant, while primarily zoonotic, showed some human-to-human transmission within households. The fact that this variant disproportionately affected children and rural populations highlighted the importance of equitable healthcare access. The delayed international response, coupled with a shortage of vaccines and medications in the most affected regions, compounded the epidemic's impact, especially in the DRC and other African nations.

Moreover, the socio-economic and cultural barriers present in many African countries have further exacerbated the challenges associated with managing Mpox outbreaks. Poor healthcare infrastructure, particularly in rural areas, has made it difficult to effectively diagnose and treat cases of Mpox. Many healthcare facilities in affected regions lack the diagnostic tools and trained personnel needed to promptly identify Mpox, leading to delays in case detection and isolation. In addition, cultural practices and reliance on traditional healers can contribute to delays in seeking medical care, allowing the virus to spread more widely before it is controlled [19]. Social stigma and mistrust of public health authorities also contribute to underreporting of cases, which hampers efforts to track the spread of the virus and implement effective containment measures [76].

Taken together, these factors—waning immunity from the cessation of smallpox vaccination, increased human–animal interactions driven by environmental changes, socio-economic barriers, and global mobility—have combined to create an environment in which Mpox outbreaks have become more frequent and severe. WHO has emphasized the need for stronger surveillance systems, particularly in rural areas, to detect cases early and prevent widespread transmission. Additionally, the organization has called for increased investment in healthcare infrastructure in affected countries, where limited resources and healthcare access continue to impede effective outbreak management. WHO's guidance also stresses the importance of addressing environmental factors, such as deforestation and human encroachment into wildlife habitats, which are contributing to the increased frequency of zoonotic spillovers [92]. In light of these distinct transmission patterns and demographic shifts, WHO has continued to advocate for differentiated approaches to outbreak management tailored to the specific characteristics of each variant and the populations affected. This includes ongoing vaccination efforts, particularly in regions like the DRC, and increased support for research into the long-term efficacy of vaccines and treatments against emerging Mpox variants.

## 4 Future challenges and considerations for curbing Mpox outbreak in Africa

The ongoing struggle to manage and control Mpox outbreaks in Africa can be attributed to a complex interplay of challenges, ranging from weak public health infrastructure [19] to environmental and ecological challenges [23]. These challenges not only complicate efforts to contain the disease but also contribute to its persistence and spread across the continent. Understanding these complexities of each challenge is crucial for developing effective strategies to combat Mpox and prevent future outbreaks.

### 4.1 Weak public health infrastructure

One of the most significant factors impeding the management of Mpox outbreaks in African countries is the weak public health infrastructure [18]. In many African nations, healthcare systems are under-resourced and underdeveloped, particularly in rural and remote areas where Mpox is most likely to emerge. Inadequate healthcare facilities, limited diagnostic capacity, and a severe shortage of trained healthcare professionals are common challenges that hinder the effective surveillance, diagnosis, and treatment of Mpox cases [19]. In regions with weak health infrastructure, delays in detecting and diagnosing Mpox can lead to the uncontrolled spread of the disease [28]. The lack of advanced laboratory facilities means that healthcare providers often rely on clinical diagnosis, which can be difficult due to the similarity of Mpox symptoms to those of other febrile illnesses, such as chickenpox or measles [93]. This diagnostic uncertainty, combined with insufficient access to necessary medical supplies and isolation facilities, hampers the timely implementation of control measures, such as patient isolation and contact tracing [23]. As a result, outbreaks can quickly escalate, overwhelming the fragile healthcare systems. The shortage of healthcare professionals trained to recognize and manage Mpox exacerbates the situation. In many cases, frontline healthcare workers may lack the knowledge and resources to identify and respond to Mpox effectively, leading to misdiagnoses, inappropriate treatment, and further spreading of the virus [94]. Moreover, the limited availability of personal protective equipment (PPE) and other critical resources in healthcare settings increases the risk of healthcare-associated transmission, putting both patients and healthcare workers at risk.

### 4.2 Lack of surveillance and reporting systems

Effective management of infectious diseases like Mpox depends heavily on robust surveillance and reporting systems [8,95], which are often lacking in many African countries. The absence of comprehensive systems to detect, report, and monitor Mpox cases in real time poses a significant challenge to controlling outbreaks [46]. Without timely and accurate data, public health authorities are unable to track the spread of the disease, identify emerging hotspots, and deploy targeted interventions to prevent further transmission. The lack of surveillance infrastructure is compounded by the underreporting of Mpox cases [96], which is often driven by stigma, fear, and lack of awareness among affected populations. In some communities, individuals may be reluctant to report symptoms or seek medical care due to concerns about social ostracization [76,97] or the belief that traditional healers can provide more effective treatment. This underreporting not only skews the true epidemiological picture of Mpox but also delays the public health response, allowing the disease to spread unchecked. Furthermore, the integration of surveillance data across regions and countries is often inadequate, leading to gaps in the understanding of Mpox transmission dynamics [19]. This lack of coordination between local, national, and regional health authorities hinders the development of comprehensive response strategies and weakens the overall capacity to manage outbreaks effectively.

### 4.3 Socio-economic and cultural factors

Socio-economic and cultural factors also play a pivotal role in the challenges associated with managing Mpox in Africa [63]. Poverty, inadequate access to healthcare, and low levels of education are prevalent in many of the regions most affected by Mpox, creating an environment where the disease can easily spread [19]. In impoverished communities, limited access to healthcare services means that individuals may not seek medical care until the disease has progressed, increasing the risk of transmission within households and communities. Cultural beliefs and practices further complicate the management of Mpox. In many African societies, there is a strong reliance on traditional healers for treating illnesses, including Mpox [98]. While traditional medicine plays an important role in the cultural and spiritual life of these communities, it can delay the seeking of formal medical care, leading to worse health outcomes and increased transmission of the virus. Additionally, practices involving close contact with animals, such as hunting, butchering, and consuming bushmeat, are deeply ingrained in many communities [23]. These practices increase the risk of zoonotic transmission of the MPXV from animals to humans, especially in regions where the virus is endemic in wildlife populations. Education and awareness campaigns are often limited, resulting in a lack of understanding about the transmission and prevention of Mpox [65]. Misconceptions about the disease, combined with low health literacy, can lead to fear, stigma, and inappropriate responses to outbreaks, further hindering efforts to control the spread of the virus.

### 4.4 Limited access to vaccines and therapeutics

A critical challenge in controlling Mpox outbreaks in Africa is the limited access to vaccines and therapeutics [4]. The smallpox vaccine, which offers cross-protection against Mpox, has not been widely available since the global eradication of smallpox in 1980 [42]. As a result, a large portion of the population is susceptible to Mpox, particularly in regions where the virus is endemic. The absence of routine smallpox vaccination programs has left many communities without the necessary immunity to prevent the spread of Mpox. Moreover, the availability of specific vaccines and antiviral treatments for Mpox remains limited in many African countries [23]. Although newer vaccines, such as the Modified Vaccinia Ankara (MVA) vaccine, have been developed and are effective against Mpox [99], the uneven and insufficient distribution of vaccines in Africa hinders effective vaccination campaigns during outbreaks, particularly affecting high-risk groups like healthcare workers and individuals in endemic areas. The scarcity of antiviral treatments further complicates the clinical management of Mpox cases [100]. Without effective treatments, healthcare providers depend on supportive care, which may be inadequate to prevent complications or lessen disease severity. This lack of therapeutic options places additional pressure on healthcare systems, leading to longer hospital stays and increased resource strain.

### 4.5 Environmental and ecological factors

Environmental and ecological factors significantly contribute to the persistence of Mpox outbreaks in Africa [101]. Human activities such as deforestation, agricultural expansion, and urbanization have led to increased encroachment into wildlife habitats, disrupting ecosystems and bringing humans into closer contact with animals that serve as reservoirs for the MPXV [23]. This increased human–animal interaction heightens the risk of zoonotic spillover events, where the virus is transmitted from animals to humans. Deforestation has had a profound impact on the transmission dynamics of Mpox [23]. Deforestation for agriculture and logging drives wildlife species that carry the MPXV, like rodents and primates, closer to human populations. This raises the chances of virus transmission through contact with infected animals or contaminated surroundings [52]. Additionally, the loss of biodiversity and the alteration of natural habitats can disrupt predator-prey relationships, potentially leading to an increase in the population of reservoir species and, consequently, a higher risk of zoonotic transmission. Climate change is another factor that may influence the spread of Mpox [23]. Changes in temperature, rainfall patterns, and other climatic factors can alter the distribution and behavior of wildlife species, potentially expanding the geographic range of the MPXV [102]. As climate change alters habitats, Mpox may appear in new regions where it was not previously found, complicating surveillance and control efforts. Furthermore, the proximity of human settlements to forested areas where the virus is endemic heightens the risk of transmission, particularly in rural communities that rely on forest resources for their livelihoods [64]. The cultural and economic importance of activities such as hunting and bushmeat consumption in these communities means that people frequently meet potential reservoirs of the virus, increasing the risk of zoonotic transmission [52]. Table 3 outlines a detailed description of the key factors contributing to Mpox challenges in Africa and their impact on Mpox management.

**Table 3:** Key factors contributing to persistent Mpox challenges in Africa.

Factor	Description	Impact on Mpox management
Weak public health infrastructure	Inadequate healthcare facilities, limited diagnostic capacity due to insufficient medical supplies, shortage of trained and qualified medical personnel and limited availability of PPE and other critical resources [72,103]	Delays in detecting and diagnosing Mpox leading to uncontrolled spread, difficulty in patient isolation and overwhelmed healthcare systems in events of large outbreaks [72,103,104]
Lack of surveillance and reporting systems	Absence of real-time detection, reporting and monitoring of Mpox cases, under reporting due to limited medical infrastructure and poor coordination among health authorities [66,72,103])	Inability to track Mpox spread effectively, poor outbreak containments and high rates of unreported transmission and mortality [72,103]
Socio-economic and cultural factors	High levels of poverty, limited access to healthcare and patronage of traditional healers in rural areas, cultural practice such as hunting, butchering and consuming of exotic bushmeat [66,104]	Low literacy levels about Mpox, reliance on traditional practices may facilitate human-to-human transmission and enhanced risk of zoonotic transmission from wildlife to humans [66,104]
Limited access to vaccines and therapeutics	Insufficient availability of smallpox vaccine, limited availability of novel vaccines, i.e. MVA, logistics issues in the distribution of available vaccine, funding shortages to develop vaccine for local communities and hesitancy and poor reception to vaccine [103]	Difficulty in implementing effective vaccination campaigns and limited vaccine coverage in affected areas [66]
Environmental and ecological factors	Deforestation, agricultural expansion and urbanisation, climate change and loser interactions between human and wildlife [103,104]	Increased risk of new zoonotic spillover infections due to exposure of new and previously unexposed human population, and change in environment complicates the prediction and prevention of new outbreaks [76,104]

Abbreviations: PPE, personal protective equipment; MVA, Modified Vaccinia Ankara.

## 5 Recommendations and future directions

Effectively managing and preventing future Mpox outbreaks requires a multifaceted approach that addresses the diverse factors contributing to the spread and persistence of the virus. The following recommendations outline key actions that must be taken to strengthen public health systems, improve disease surveillance, and foster global collaboration. These recommendations are aligned with the distinct phases of Mpox outbreaks, taking into account changes in transmission dynamics and the ongoing evolution of the virus [32,105].

### 5.1 Strengthening public health infrastructure

Investment in public health infrastructure is essential to building the capacity of healthcare systems to effectively respond to Mpox outbreaks. Strengthening diagnostic capabilities is critical for early and accurate detection, enabling rapid outbreak containment [106]. This includes equipping laboratories with advanced tools for Mpox detection and training healthcare workers in the diagnosis and treatment of the disease [107]. In underserved rural areas, improving healthcare facilities, staffing, and ensuring adequate medical supplies will be fundamental to mitigating the impact of Mpox and other emerging infectious diseases. By enhancing healthcare infrastructure, countries can better manage outbreaks and prevent wider transmission.

### 5.2 Enhancing surveillance and reporting

A robust surveillance and reporting system is vital for the timely detection and management of Mpox outbreaks. Implementing comprehensive surveillance systems, including community-based reporting, can significantly improve case detection and tracking [12,18]. These systems should capture real-time data, enabling rapid public health responses to emerging outbreaks [108]. Digital tools, such as mobile health technologies, can be incorporated to streamline data collection and analysis, improving overall outbreak response. Furthermore, public health authorities should prioritize public education campaigns that reduce stigma and encourage the timely reporting of suspected cases, thereby enhancing surveillance effectiveness [108].

### 5.3 Improving access to vaccines and therapeutics

Ensuring equitable access to vaccines and therapeutics is critical to controlling Mpox outbreaks. Increasing global production of the MVA vaccine is necessary to meet the demand during outbreaks, particularly in endemic regions [3]. Equitable distribution of vaccines to high-risk populations, including healthcare workers and communities in close contact with wildlife, is essential for reducing transmission. In addition to existing vaccines, the reintroduction of smallpox vaccination for high-risk groups may be considered, given its cross-protection against Mpox [109]. Simultaneously, research into the development of new therapeutics and the repurposing of existing antiviral treatments must be prioritized to manage severe cases and make treatments widely accessible, regardless of economic status or location [109].

### 5.4 Addressing socio-economic and cultural barriers

Socio-economic and cultural factors play a significant role in Mpox transmission, particularly in regions where traditional practices and limited healthcare access hinder early detection and treatment [10]. Public health interventions should focus on educating communities about the risks associated with hunting and consuming bushmeat and promoting safer alternatives [66]. Additionally, improving access to healthcare in affected regions, lowering financial barriers, and enhancing transportation infrastructure are necessary steps toward overcoming these barriers. Addressing poverty and improving living conditions are long-term goals that will contribute to more effective disease management and reduced transmission rates.

### 5.5 Environmental and ecological management

Environmental and ecological factors are key determinants of zoonotic disease transmission, including Mpox [23]. Protecting wildlife habitats and reducing deforestation are critical to minimizing human–wildlife interactions that can lead to zoonotic spillovers. Incorporating environmental conservation into public health strategies can mitigate these risks. Sustainable land-use practices, biodiversity conservation, and reforestation projects are essential for managing human–wildlife interactions and reducing transmission risk. Public health and environmental agencies must collaborate to develop policies that balance economic development with ecosystem preservation [23].

### 5.6 Global collaboration and support

Managing Mpox outbreaks requires strong international cooperation [32,46]. Global health organizations, governments, and non-governmental organizations (NGOs) should collaborate to provide financial, technical, and research support to countries heavily impacted by Mpox. This includes enhancing healthcare infrastructure, improving surveillance systems, and ensuring equitable access to vaccines and treatments. Through international partnerships, research on new vaccines and treatments can be accelerated, and public health strategies can be optimized to combat Mpox and other emerging diseases.

### 5.7 Genetic evolution of Mpox and phase-specific prevention

Understanding the genetic evolution of the MPXV is critical for future prevention and control measures. Genetic mutations, such as those identified in MPXVgp012 and MPXVgp191, have potentially enhanced human-to-human transmission, as seen in recent outbreaks [110,111]. Continuous monitoring of these mutations is crucial for predicting shifts in transmission dynamics and disease severity [112]. Additionally, the virus's open pan-genome and ongoing evolution, driven by gene turnover, underscore the importance of genomic surveillance in tracking Mpox's adaptability [113].

Phase-specific prevention strategies are essential to addressing the evolving nature of Mpox outbreaks. Vaccination efforts should be tailored to different outbreak phases, focusing on high-risk populations during human-to-human transmission phases, such as the MSM-driven outbreak in 2022–2024 [88]. In zoonotic phases, vaccination strategies should target individuals in frequent contact with wildlife, such as hunters and agricultural workers in rural Africa. Surveillance and containment efforts must also be adapted to each phase, with digital tools prioritized in urban settings and traditional, community-based surveillance strengthened in rural areas [92].

The One Health approach must be fully integrated into future Mpox control efforts, acknowledging the interconnectedness of human, animal, and environmental health. Collaborative efforts between public health, veterinary, and environmental agencies are essential for addressing the root causes of zoonotic transmission, particularly in regions experiencing deforestation or environmental degradation. Preventive measures should include habitat conservation and sustainable land-use practices, alongside public health interventions that promote safe interactions with wildlife and discourage bushmeat consumption. By adopting these integrated, phase-specific strategies, public health authorities can more effectively manage future Mpox outbreaks and reduce the global burden of the disease.

## 6 Conclusion

The ongoing management of Mpox outbreaks in Africa highlights various public health, socio-economic, cultural, and environmental challenges. The current outbreak has revealed significant deficiencies in global preparedness and access to vital resources like vaccines and treatments. Urgent action is needed to close these gaps to prevent future outbreaks from becoming severe public health crises. Learning from past experiences is crucial for better management of emerging infectious diseases. Recommended strategies include strengthening public health infrastructure, enhancing surveillance and reporting, improving access to vaccines and treatments, addressing socio-economic and cultural barriers, and managing environmental risks. African countries must build resilience against future outbreaks, and global collaboration is essential for the success and sustainability of these efforts. Ignoring these issues could lead to a more serious public health crisis both in Africa and globally. It is crucial for global action to close existing gaps and create a more equitable and coordinated response to emerging infectious diseases, like Mpox. This proactive strategy is essential for protecting public health and vulnerable populations from future threats.

## CRediT authorship contribution statement

**David B. Olawade:** Writing – review & editing, Writing – original draft, Resources, Project administration, Methodology, Investigation, Conceptualization. **Ojima Z. Wada:** Writing – review & editing, Writing – original draft, Project administration, Methodology, Formal analysis. **Sandra Chinaza Fidelis:** Writing – review & editing, Writing – original draft. **Oluwafemi S. Oluwole:** Writing – review & editing, Writing – original draft. **Chibuike S. Alisi:** Writing – review & editing, Writing – original draft. **Nifemi F. Orimabuyaku:** Writing – review & editing, Writing – original draft. **Aanuoluwapo Clement David-Olawade:** Writing – review & editing, Writing – original draft, Supervision.

## Funding sources

This review paper did not receive any specific grant from funding agencies in the public, commercial or not-for-profit sectors.

## Declaration of competing interest

The authors declare that there is no conflict of interest to disclose.
